# Modeling inflation rates and exchange rates in Ghana: application of multivariate GARCH models

**DOI:** 10.1186/s40064-015-0837-6

**Published:** 2015-02-24

**Authors:** Ezekiel NN Nortey, Delali D Ngoh, Kwabena Doku-Amponsah, Kenneth Ofori-Boateng

**Affiliations:** Department of Statistics, University of Ghana, P. O. Box LG 115, Legon-Accra, Ghana; Ghana Institute of Management and Public Administration Business School, Achimota-Accra, Ghana

**Keywords:** DCC, BEKK, GARCH, Ghana, Volatility, Inflation, Exchange, Interest rates

## Abstract

This paper was aimed at investigating the volatility and conditional relationship among inflation rates, exchange rates and interest rates as well as to construct a model using multivariate GARCH DCC and BEKK models using Ghana data from January 1990 to December 2013. The study revealed that the cumulative depreciation of the cedi to the US dollar from 1990 to 2013 is 7,010.2% and the yearly weighted depreciation of the cedi to the US dollar for the period is 20.4%. There was evidence that, the fact that inflation rate was stable, does not mean that exchange rates and interest rates are expected to be stable. Rather, when the cedi performs well on the forex, inflation rates and interest rates react positively and become stable in the long run. The BEKK model is robust to modelling and forecasting volatility of inflation rates, exchange rates and interest rates. The DCC model is robust to model the conditional and unconditional correlation among inflation rates, exchange rates and interest rates. The BEKK model, which forecasted high exchange rate volatility for the year 2014, is very robust for modelling the exchange rates in Ghana. The mean equation of the DCC model is also robust to forecast inflation rates in Ghana.

## Introduction

When the general level of prices is relatively stable, the uncertainties of time-related activities such as investment diminish. This helps to promote full employment and strong economic growth. When price stability is achieved and maintained, monetary policy makers have done their job well (Sobel et al. 2006). Conceivably, one of the most important responsibilities of every government is fostering a healthy economy, which benefits all her citizens. The government through its ability to tax, spend and control money supply, attempts to promote full employment, price stability and economic growth.

The importance of price stability is also emphasized in the Maastricht agreement, which defined the framework for a single European Currency, Euro, and identified price stability as the main objective of the new European Central Bank (McEachern 2006). Deflation could result to doom for an economy; that is, it weakens consumer demand for goods and services as households are likely not to spend, believing that prices will continue to fall. This means that businesses as well as government may be unable to pay debts and could result in retrenchment. Emphasizing this point is Lagarde; the Managing Director of the IMF, in April 2014 who cautioned the euro area that, a prolonged period of “low-inflation” or deflation can suppress demand and output, and overturn growth and jobs.

According to Goldberg and Knetter (1997) exchange rate pass-through is the percentage change in local currency import prices resulting from a one percent change in the exchange rate between the exporting and importing countries. Exchange rate pass-through therefore is the effect (positive or negative) of exchange rates on import and export prices, consumer prices or inflation, investments as well as trade volumes. Engel and Rogers (1996) established that crossing the US-Canada border can considerably raise relative price volatility and that exchange rate fluctuations explain about one-third of the volatility increase. That is US-Canada border is an important determinant of relative price volatility even after making due allowance for the role of distance. Parsley and Wei **(**2001) confirmed previous findings that crossing national borders adds significantly to price dispersion.

The demand for and supply of money are the key determinants of exchange rates. Interest Rate Parity is an important concept that explains the equilibrium state of the relationship between interest rate and exchange rate of two countries. The foreign exchange market is in equilibrium when deposits of all currencies offer the same expected rate of return. The condition that the expected returns on deposits of any two currencies are equal when measured in the same currency is called the interest parity condition. It implies that potential holders of foreign currency deposits view them all as equally desirable assets, provided their expected rates of return are the same. Given that the expected return on say US dollar deposits is 4 percent greater than that on Ghana cedi deposits, all things being equal, no one will be willing to continue holding Ghana cedi deposits, and holders of Ghana cedi deposits will be trying to sell them for US dollar deposits. There will therefore be an excess supply of Ghana cedi deposits and an excess demand for US dollar deposits in the foreign exchange market (Krugman et al. 2012).

An important theory of the relationship between inflation rate and interest rate is the Fisher effect; sometimes referred to as the Fisher hypothesis by Irvin Fisher. Fisher proved mathematically that the nominal interest rate is equal to the real interest rate minus the expected (predicted) inflation rate. The Fisher effect simply explains for example that; if the nominal interest rate is say 50 per cent for a given period, and the predicted inflation rate during that same period is 20 per cent, then the real interest rate is 30 per cent. The movement in short term interest rates primarily reflects fluctuation in expected inflation, which in effect has a predictive ability for future inflation (Mishkin and Simon 1995).

The primary objective of the Central Bank of Ghana is to maintain stability in the general level of prices (Bank of Ghana Act 2002). Price Stability is, therefore, one of the most important indicators of the health of a nation’s economy. It must be noted that price stability alone might not be enough for a healthy economy.

Several studies have been conducted on modelling inflation rates in Ghana, and majority of these used the constant variance assumption model. Although Mbeah-Baiden (2013) used non-constant variance models to model inflation rates in Ghana, his work only considered a univariate analysis of inflation rates. In the developed countries where a number of the researchers have modelled financial data series using Multivariate Generalized Autoregressive Conditional Heteroscedastic (MGARCH) models, none has modelled the co-movements of inflation rates, exchange rates and interest rates. The MGARCH models have not been explored enough on Ghanaian data and to a very large extent, Africa. It must be noted that Atta-Mensah and Bawumia (2003) used Vector Error Correction forecasting model for Ghana and concluded that growth rate, broad money supply (M2+) and depreciation of exchange rate are the main drivers of higher inflation.

The main objective of the study is to investigate the volatility and conditional relationship of inflation, exchange and interest rates and to construct a model using the multivariate GARCH BEKK (Baba, Engle, Kraft and Kroner) and DCC (Dynamic Conditional Correlation) models.

A researcher can apply all these models on data series and the best model is chosen based on the performance of the model using a criterion. According to (Doan: RATS Handbook for ARCH/GARCH and Volatility Models. pp: 38. Evanston, United States: Estima, Unpublished Draft Book), the application of BEKK and DCC in modelling the conditional variance generally achieved similar results and the difference is negligible.

### Data and methodology

The monthly inflation rates, average monthly exchange rates (cedi to US dollar) and interest rates (lending rate to the public) in Ghana spanning the period January 1990 to December 2013 were used for the study. This means that a total of 288 data points were considered for each variable. The sources of data were the Ghana Statistical Service (GSS) and Ghana Commercial Bank (GCB).

The data were analyzed using multivariate GARCH, DCC and BEKK models. The procedure most often used in the model estimation involves the maximization of a likelihood function constructed on the assumption of independently and identically distributed standardized residuals.

According to Engle and Sheppard (2001), analyzing and understanding how the univariate GARCH works is fundamental for the study of the Dynamic Conditional Correlation multivariate GARCH model. The DCC model is a nonlinear combination of univariate GARCH and its matrix is based on how the univariate GARCH (1, 1) process works.

Suppose that the stochastic process ${\left\{{x}_t\right\}}_t^T$ denotes the return during a specific time period, where *x*_*t*_ is the return observed at time *t*. Assuming for instance that the model for a return is given as: *x*_*t*_ = *μ*_*t*_ + *ε*_*t*_, where *μ*_*t*_ = *Ε*(*x*_*t*_/*λ*_*t* − 1_) denotes the conditional expectation of the return series, *ε*_*t*_ is the condition error and *λ*_*t* − 1_ = *σ*(*x* : *s* ≤ *t* − 1) represent the sigma field (information set) generated by the values of the return until time *t* - 1. Suppose that the conditional errors are conditional standard deviations of the returns ${h}_t^{1/2}=Var{\left({x}_t/{\lambda}_{t-1}\right)}^{1/2}$ times is independent and identically normally distributed with zero mean and a unit variance stochastic variable *y*_*t*_. Note that *h*_*t*_ and *y*_*t*_ are independent for all time *t*, ${\varepsilon}_t=\sqrt{h_t{y}_t}\sim N\left(0,{h}_t\right)$. Lastly, assume that the conditional expectation *μ*_*t*_ = 0, which implies that ${x}_t=\sqrt{h_t{y}_t}$ and *x*_*t*_/*λ*_*t* − 1_ ∼ *N*(0, *h*_*t*_).

Conditioning of economic and financial models are mostly stated as the regression of a variable’s present values of the variable on the same variable’s past values as indicated in the GARCH(p,q) model proposed by (Bolleslev 1986) is given in equation (1):

1 $$ \begin{array}{l}{h}_t=\phi +\sum_{i=1}^p{\alpha}_i{\varepsilon}_{t-1}^2+\sum_{i=1}^q{\beta}_i{h}_{t-1}\kern1em ,\kern1em p\ge 0\kern1.12em ,\kern1em q>0\\ {}\phi \ge 0\kern0.5em ,\kern0.5em {\alpha}_i\ge 0,\kern1em i=1,2,3,\dots, p;{\beta}_i\ge 0\kern1em for\kern1em i=1,2,3,..\ .,q.\end{array} $$

The GARCH(p,q) consist of the three terms, these are:

(i) *ϕ* - the weighted long run variance
(ii) $\sum_{i=1}^p{\alpha}_i{\varepsilon}_{t-1}^2$ - the moving average term, which is the sum of the *p* previous lags of squared-innovations multiplied by the assigned weight *α*_*i*_ for each lagged square innovation
(iii) $\sum_{i=1}^q{\beta}_i{h}_{t-1}$ - the autoregressive term, which is the sum of the *q* previous lagged variances multiplied by the assigned *β*_*i*_ for each lagged variance.

Since the variance is non-negative by definition, the process ${\left\{{h}_t\right\}}_{t=0}^{\infty }$ must also be non-negative valued.

### Baba, Engle, Kraft and Kroner (BEKK) model

To ensure positive definiteness, a new parameterization of the conditional variance matrix *H*_*t*_ was defined by, (Baba, Engle, Kraft, Kroner: Multivariate simultaneous generalized ARCH at the University of California, San Diego, unpublished) and became known as the BEKK model, which is viewed as another restricted version of the VEC model. It achieves the positive definiteness of the conditional covariance by formulating the model in a way that this property is implied by the model structure. The form of the BEKK model is as:

2 $$ {H}_t=C{C}^{\hbox{'}}+\sum_{j=1}^q\sum_{k=1}^K{A}_{kj}^{\hbox{'}}{\varepsilon}_{t-j}{\varepsilon}_{{}_{t-j}}^{\hbox{'}}{A}_{kj}+\sum_{j=1}^p\sum_{k=1}^K{B}_{kj}^{\hbox{'}}{H}_{t-j}{B}_{kj} $$

where *A*_*kj*_ and *B*_*kj*_ a *N* × *N* parameter matrices, and *C* is a lower triangular matrix. The purpose of decomposing the constant term in equation (2) into a product of the two triangular matrices is to guarantee the positive semi-definiteness of *H*_*t*_. Whenever *K* > 1 an identification problem would be generated for the reason that there are not only a single parameterization that can obtain the same representation of the model.

The first-order BEKK model is given as:

3 $$ {H}_t=C{C}^{\hbox{'}}+{A}^{\hbox{'}}{\varepsilon}_{t-j}{\varepsilon}_{{}_{t-j}}^{\hbox{'}}A+{B}^{\hbox{'}}{H}_{t-j}B $$

The BEKK model specified in equation (3) also has its diagonal form by assuming that the matrices *A*_*kj*_ and *B*_*kj*_ are diagonal. It is a restrictive version of the DVEC model. The most restricted version of the diagonal BEKK is the scalar BEKK one with *A* = *aI* and *B* = *bI* where *α* and *b* are scalars.

Estimation of the BEKK model still bears large computations due to several matrix transpositions. The number of parameters of a complete BEKK model is (*p* + *q*)*KN*^2^ + *N*(*N* + 1)/2 whereas in the diagonal BEKK, the number of parameters reduces to (*p* + *q*)*KN* + *N*(*N* + 1)/2. The BEKK form is not linear in the parameters, which makes the convergence of the model difficult. However, the model structure automatically guarantees the positive definiteness of *H*_*t*_. Under the overall consideration, it is assumed that *p* = *q* = *k* = 1 in BEKK forms of application. The difference between the results of BEKK model and the DCC model is highly negligible.

### The Dynamic Conditional Correlation (DCC) Model

To extend the assumptions in the univariate GARCH to multivariate case, suppose that we have *n* assets in a portfolio and the return vector is *x*_*t*_ = (*x*_1*t*_, *x*_2*t*_, *x*_3*t*_, …, *x*_*nt*_) '. Furthermore, assume that the conditional returns are normally distributed with zero mean and conditional covariance matrix ${H}_t=E\left\{{x}_t{x}_t^{,}/{\lambda}_{t-1}\right\}$. This implies that ${x}_t={H}_t^{1/2}{y}_t$ and *x*_*t*_/*λ*_*t* − 1_ ∼ *N*(0, *H*_*t*_), *z*_*t*_ = (*z*_1*t*_, *z*_2*t*_, *z*_3*t*_, …, *z*_*nt*_) ' ∼ *N*(0, *I*_*n*_) and *I*_*n*_ is the identity matrix of order n. ${H}_t^{1/2}$ may be obtained by Cholesky decomposition of *H*_*t*_.

In DCC-model, the covariance matrix is decomposed into *H*_*t*_ ≡ *D*_*t*_*X*_*t*_*D*_*t*_, where *D*_*t*_ is the diagonal matrix of time varying standard variation from univariate GARCH process

$$ {D}_t=\left[\begin{array}{cccc}\hfill \sqrt{h_{1t}}\hfill & \hfill 0\hfill & \hfill \cdots \hfill & \hfill 0\hfill \\ {}\hfill 0\hfill & \hfill \sqrt{h_{2t}}\hfill & \hfill \cdots \hfill & \hfill 0\hfill \\ {}\hfill \vdots \hfill & \hfill \vdots \hfill & \hfill \ddots \hfill & \hfill \vdots \hfill \\ {}\hfill 0\hfill & \hfill 0\hfill & \hfill \cdots \hfill & \hfill \sqrt{h_{nt}}\hfill \end{array}\right]. $$

The specification of elements in the *D*_*t*_ matrix is not only restricted to the GARCH(p,q) described in equation (1) but to any GARCH process with normally distributed errors which meet the requirements for suitable stationary and non-negative conditions. The number of lags for each assets and series do not need to be the same either. However, *X*_*t*_ is the conditional correlation matrix of the standardized disturbances *ε*_*t*_; where

$$ {X}_t=\left[\begin{array}{cccc}\hfill 1\hfill & \hfill {Q}_{12,t}\hfill & \hfill \cdots \hfill & \hfill {Q}_{1n,t}\hfill \\ {}\hfill {Q}_{21,t}\hfill & \hfill 1\hfill & \hfill \cdots \hfill & \hfill {Q}_{2n,t}\hfill \\ {}\hfill \vdots \hfill & \hfill \vdots \hfill & \hfill \ddots \hfill & \hfill \vdots \hfill \\ {}\hfill {Q}_{n1,t}\hfill & \hfill {Q}_{n2,t}\hfill & \hfill \cdots \hfill & \hfill 1\hfill \end{array}\right]\kern0.36em \mathrm{and}\kern0.36em {\varepsilon}_t={D}_t^{-1}{x}_t\sim N\left(0,{X}_t\right). $$

Thus, the conditional correlation is the conditional covariance between the standardized disturbances. By the definition of the covariance matrix, *H*_*t*_ has to be positive definite. Since *H*_*t*_ is a quadratic form based on *X*_*ts*_, it follows from basics in linear algebra that *X*_*t*_ has to be positive definite to ensure that *H*_*t*_ is positive definite. By the definition of the conditional correlation matrix all the elements have to be equal to or less than one. To ensure that all of these requirements are met, *X*_*t*_ is decomposed into ${X}_t={Q}_t^{*-1}{Q}_t{Q}_t^{*-1}$, where *Q*_*t*_ is a positive definite matrix defining the structure of the dynamics and ${Q}_t^{*-1}$ rescales the elements in *Q*_*t*_ to ensure that |*q*_*ij*_| ≤ 1. This implies that, ${Q}_t^{*-1}$ is simply the inverted diagonal matrix with the squared root diagonal elements of *Q*_*t*_.

$$ {Q}_t^{*-1}=\left[\begin{array}{cccc}\hfill 1/\sqrt{Q_{11t}}\hfill & \hfill 0\hfill & \hfill \cdots \hfill & \hfill 0\hfill \\ {}\hfill 0\hfill & \hfill 1/\sqrt{Q_{11t}}\hfill & \hfill \cdots \hfill & \hfill 0\hfill \\ {}\hfill \vdots \hfill & \hfill \vdots \hfill & \hfill \ddots \hfill & \hfill 0\hfill \\ {}\hfill 0\hfill & \hfill 0\hfill & \hfill \cdots \hfill & \hfill 1/\sqrt{Q_{11t}}\hfill \end{array}\right]. $$

Suppose that *Q*_*t*_ has the following dynamics:

4 $$ {Q}_t=\left(1-\alpha -\beta \right)\overline{Q}+\alpha {\varepsilon}_{t-1}{\varepsilon}_{t-1}^{,}+\beta {Q}_{t-1} $$

where $\overline{Q}$ is the unconditional covariance of the standardized disturbances $\overline{Q}=\operatorname{cov}\left({\varepsilon}_t{\varepsilon}_t^{,}\right)=E\left\{{\varepsilon}_t{\varepsilon}_t^{,}\right\}$, and *β* are scalars.

The dynamic structure defined above is the simplest multivariate GARCH called Scalar GARCH. A major caveat of this structure is the all correlations obey the same structure.

The structure can be extended to the general DCC(P,Q)

5 $$ {Q}_t=\left(1-{\displaystyle \underset{i=1}{\overset{P}{\varSigma }}}{\alpha}_i-{\displaystyle \underset{j=1}{\overset{Q}{\varSigma }}}{\beta}_j\right)\overline{Q}+{\displaystyle \underset{i=1}{\overset{P}{\varSigma }}}{\alpha}_i{\varepsilon}_{t-1}{\varepsilon}_{t-1}^{,}+{\displaystyle \underset{j=1}{\overset{Q}{\varSigma }}}{\beta}_j{Q}_{t-1} $$

In this work, only the DCC(1,1) will be utilized.

### Constraints of the DCC(1,1) Model

If the covariance matrix is not positive definite then it is impossible to invert the covariance matrix which is essential in portfolio optimization. To guarantee a positive definite *H*_*t*_ for all *t*, simple conditions on the parameters are imposed. Firstly, the conditions for the univariate GARCH model has to be satisfied. Similar conditions on the dynamic correlations are also required, namely: *β* ≥ 0 and *α* ≥ 0, *α* + *β* < 1, *Q*_0_ has to be positive definite.

### Estimation of the DCC(1,1) Model

In order to estimate the parameters of *H*_*t*_, that is to say *θ* = (*θ*_1_, *θ*_2_), the following log-likelihood function *ℓ* can be used when the errors are assumed to be multivariate normally distributed:

6 $$ \begin{array}{l}\ell \left(\theta \right)=-\frac{1}{2}\sum_{t=1}^T\left(n \log \left(2\pi \right)+ \log \left(\left|{H}_t\right|\right)+{x}_t^{\hbox{'}}{H}_t^{-1}{x}_t\right)\\ {}\ell \left(\theta \right)=-\frac{1}{2}\sum_{t=1}^T\left(n \log \left(2\pi \right)+ \log \left(\left|{D}_t{X}_t{D}_t\right|\right)+{x}_t^{\hbox{'}}{D}_t^{-1}{X}_t^{-1}{D}_t^{-1}{x}_t\right)\\ {}\ell \left(\theta \right)=-\frac{1}{2}\sum_{t=1}^T\left(n \log \left(2\pi \right)+2 \log \left(\left|{D}_t\right|\right)+ \log \left(\left|{X}_t\right|\right)+{\varepsilon}_t^{\hbox{'}}{X}_t^{-1}{\varepsilon}_t\right)\end{array} $$

The parameters in the DCC(1,1) model specified in equation (6) can be divided into two groups, that is:

7 $$ {\theta}_1=\left({\phi}_1,{\alpha}_1,{\beta}_1,{\phi}_2,{\alpha}_2,{\beta}_2\dots, {\phi}_n,{\alpha}_n,{\beta}_n\right)\kern0.24em  and\kern0.24em {\theta}_2=\left(\eta, \psi \right). $$

The estimation follows the following two steps.

### Step one

The *X*_*t*_ matrix in the log-likelihood function is replaced with the identity matrix *I*_*n*_, which gives the following log-likelihood function specified in equation (8):

8 $$ \begin{array}{l}\ell \left({\theta}_1/{x}_t\right)=-\frac{1}{2}\sum_{t=1}^T\left(n \log \left(2\pi \right)+2 \log \left(\left|{D}_t\right|\right)+ \log \left(\left|{I}_n\right|\right)+{x}_t^{\hbox{'}}{D}_t^{-1}{I}_n{D}_t^{-1}{x}_t\right)\\ {}\ell \left({\theta}_1/{x}_t\right)=-\frac{1}{2}\sum_{t=1}^T\left(n \log \left(2\pi \right)+2 \log \left(\left|{D}_t\right|\right)+{x}_t^{\hbox{'}}{D}_t^{-1}{D}_t^{-1}{x}_t\right)\\ {}\ell \left({\theta}_1/{x}_t\right)=-\frac{1}{2}\sum_{t=1}^T\sum_{i=1}^n\left( \log \left(2\pi \right)+ \log \left({h}_{it}\right)+\frac{r_{it}^2}{h_{it}}\right)\\ {}\ell \left({\theta}_1/{x}_t\right)=-\frac{1}{2}\sum_{i=1}^n\left(T \log \left(2\pi \right)+\sum_{t=1}^T\left\{ \log \left({h}_{it}\right)+\frac{r_{it}^2}{h_{it}}\right\}\right)\end{array} $$

It is obvious that this quasi-likelihood function is the sum of the univariate GARCH log-likelihood functions. Therefore, one can use the algorithm to estimate parameter *θ*_1_ = (*ϕ*_1_, *α*_1_, *β*_1_, *ϕ*_2_, *α*_2_, *β*_2_ …, *ϕ*_*n*_, *α*_*n*_, *β*_*n*_) for each univariate GARCH process. Since the variance *h*_*it*_ for asset *i* = 1, 2, 3, … *n* is estimated for *t* ∈ {1, *T*}, then also the element of the D_t_ matrix under the same time period is estimated.

### Step two

In the second step, the correctly specified log-likelihood function is used to estimate *θ*_2_ = (*η*, *ψ*) given the estimated parameters ${\widehat{\theta}}_1=\left({\widehat{\phi}}_1,{\widehat{\alpha}}_1,{\widehat{\beta}}_1,{\widehat{\phi}}_2,{\widehat{\alpha}}_2,{\widehat{\beta}}_2\dots, {\widehat{\phi}}_n,{\widehat{\alpha}}_n,{\widehat{\beta}}_n\right)$ from step one, we obtain:

9 $$ {\ell}_2\left({\theta}_2/{\widehat{\theta}}_1,{x}_t\right)=-\frac{1}{2}\sum_{t=1}^T\left(n \log \left(2\pi \right)+2 \log \left(\left|{D}_t\right|\right)+ \log \left(\left|{X}_t\right|\right)+{\varepsilon}_t^{\hbox{'}}{X}_t^{-1}{\varepsilon}_t\right) $$

From equation (9), the first two terms in the log-likelihood are constants therefore, the two last terms including *X*_*t*_ is of interest to maximize. Hence we obtain:

10 $$ {\ell}_2\propto \log \left(\left|{X}_t\right|\right)+{\varepsilon}_t^{\hbox{'}}{X}_t^{-1}{\varepsilon}_t. $$

$\overline{Q}$ is estimated as: $\widehat{Q} = \frac{1}{T}\sum_{t=1}^T{\varepsilon}_t^{\hbox{'}}{\varepsilon}_t$.

Variance targeting is used in the dynamic structure and therefore ${\widehat{Q}}_0={\varepsilon}_0^{\hbox{'}}{\varepsilon}_0$ and since the conditional correlation matrix also is the covariance matrix of the standardized residuals, ${\widehat{X}}_0={\varepsilon}_0^{\hbox{'}}{\varepsilon}_0$.

## Results and discussion

**Figure 1:**
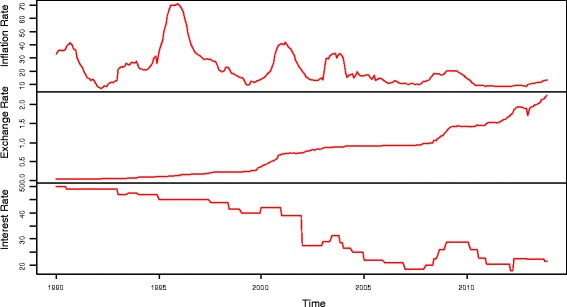
**Time series plot of inflation, exchange and interest rates from 1990 to 2013.**

Figure 1 shows the time series plot for inflation rates, exchange rates and interest rates from 1990 to 2013 based on R output. The inflation rates and interest rates plots exhibits downward trend with fluctuations, contrarily, the exchange rates plot exhibit continuous upwards trend. The movements of the plots indicate that the mean and the variance of the exchange rates data are changing overtime. This means that the mean is non constant and the variance is unstable.

**Figure 2:**
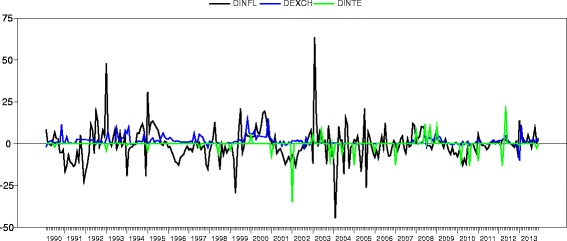
**Time series plot of natural logarithm of monthly inflation rate exchange rate and interest rate in Ghana from 1990 to 2013.**

Figure 2 displays the time series plot of the natural logarithm of inflation rates, exchange rates and interest rates from January 1990 to December 2013 using RATS 8.3. The time series plot appears to be stable after the transformation using the natural logarithm of inflation, exchange and interest rates. This suggests that the mean and variance are stable over time implying that the variables achieve stationarity after taking the natural logarithm.

The cumulative depreciation of the cedi to the US dollar from 1990 to 2013 is 7,010.2% and the yearly weighted depreciation of the cedi to the US dollar is 20.4% using the formulae in equation (11) and (12) respectively;

11 $$ \mathrm{Depreciation}=\frac{\mathrm{rat}{\mathrm{e}}_{\mathrm{e}\mathrm{nd}}-\mathrm{rat}{\mathrm{e}}_{\mathrm{initial}}}{\mathrm{rat}{\mathrm{e}}_{\mathrm{initial}}}\times 100. $$

where *n* is the number of years.

### Multivariate-GARCH modeling

Multivariate GARCH models are estimated by the quasi maximum likelihood technique. Regression Analysis of Time Series (RATS) 8.3 is a widely used software in estimating MGARCH models as a result of its flexible maximum likelihood estimation capabilities and has advantages over many other software packages on estimating MGARCH models. The optimization algorithm used for the maximum likelihood estimation in RATS is BFGS; proposed independently by Broyden (1970), Fletcher (1970), Goldfarb (1970) and Shanno (1970), (Estima 2013). This optimization algorithm uses iteration routines to obtain the coefficient estimation. As such, convergence is assumed to occur if the change in the coefficient to be estimated is less than the criterion option 0.00001 specified. RATS was used in estimating the MGARCH models for this study.

Table 1 shows both the DCC and BEKK with respective p-values of 0.99659 and 0.9869. The p-values are greater than a significance level of 0.05, hence it can be concluded that the there is no multivariate ARCH effect. This also suggests that the conditional distribution of the white noise is near Gaussian.

**Table 1:** **Test of multivariate ARCH effect and serial correlation of DCC and BEKK**

	**DCC**	**BEKK**
Lag	10	10
D.F	360	360
Stats	291.6	303.07
P-value	0.99659	0.98679

### DCC model

The estimated DCC model’s unconditional covariance matrix is given in equation (12):

12 $$ \begin{array}{l}{\mathrm{h}}_{11\mathrm{t}}=48.7058399+0.2821624{\upmu}_{1,\mathrm{t}\hbox{-} 1}^2+0.0410249{\mathrm{h}}_{11\mathrm{t}\hbox{-} 1}\\ {}{\mathrm{h}}_{22\mathrm{t}}=1.5122533+0.23933668{\upmu}_{2,\mathrm{t}\hbox{-} 1}^2+0.48564696{\mathrm{h}}_{22\mathrm{t}\hbox{-} 1}\\ {}{\mathrm{h}}_{33\mathrm{t}}=3.82780228+0.0107313{\upmu}_{3,\mathrm{t}\hbox{-} 1}^2+0.70807305{\mathrm{h}}_{33\mathrm{t}\hbox{-} 1}\\ {}{\mathrm{Q}}_{\mathrm{t}}=\left(1-0.01007687-0.9705411\right)\;\overline{\mathrm{Q}}+0.01007687{\upvarepsilon}_{\mathrm{t}\hbox{-} 1}\upvarepsilon {'}_{\mathrm{t}\hbox{-} 1}+0.9705411{Q}_{t-1}\\ {}\overline{\mathrm{Q}}=\left(\begin{array}{ccc}\hfill 1.00000\hfill & \hfill -0.03357\hfill & \hfill 0.03980\hfill \\ {}\hfill -0.03357\hfill & \hfill 1.00000\hfill & \hfill -0.86917\hfill \\ {}\hfill 0.03980\hfill & \hfill -0.86917\hfill & \hfill 1.00000\hfill \end{array}\right)\end{array} $$

**Figure 3:**
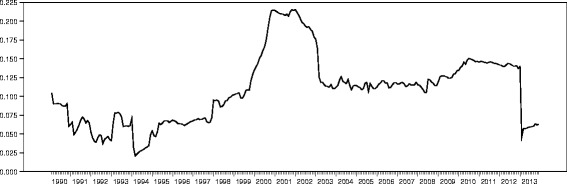
**Time series plot of the conditional correlation of inflation and exchange rates from 1990 to 2013.**

Figure 3 displays the conditional correlation between inflation rates and exchange rates from 1990 to 2013. The plot indicates that there is a positive conditional association between inflation and exchange rate. This implies that, as the local currency; the cedi depreciates to the US dollar, general levels of prices in Ghana also increases. The relationship was relatively stronger in 1991 and 1993 compared to 1992, the year election was held. The period of 1995, 1996 and 1997 as well as the years between 2003 and 2009 exhibited relatively weak correlation. Contrary, the period between 2000 and 2002 exhibited the strongest positive relationship. Depreciation of the cedi means that the cedi buys less than the US dollar, therefore, imports are more expensive and exports are cheaper. The positive relationship in the exchange rate depreciation and inflation rate means that, imported goods and services become more expensive and this affects the health of the economy especially because Ghana depends heavily on imported goods. The relationship exhibited is disincentive to cutting cost for companies whose raw materials are imported, this implies that depreciation causes cost-push inflation in the long run.

Table 2 displays seven months out-of-sample forecast of inflation rate for 2014 using the mean equation of the DCC model. The forecasts, compared to the observed rates declared by the Ghana Statistical Service indicate that there is evidence that the mean equation of the DCC model is robust in predicting inflation rate in the medium to short term. The widening of the error with time is an indication that general prices of goods and services react to the depreciation of the cedi or volatility in the exchange rate in the long run. Based on the DCC model, the mean equation is given as

13 $$ Inflatio{n}_t=48.7058399-0.12070302t $$

**Table 2:** **Seven months of 2014 out-sample forecast of inflation rate from the mean equation of the DCC model**

**Month/Year**	**Observed (%)**	**Forecast (%)**	**Error (%)**
Jan-2014	13.80	13.82	0.02
Feb-2014	14.00	13.70	−0.30
Mar-2014	14.50	13.58	−0.92
Apr-2014	14.70	13.46	−1.24
May-2014	14.80	13.22	−1.58
Jun-2014	15.00	13.10	−1.90
Jul-2014	15.30	12.98	−2.32
Aug-2014	15.90	12.86	−3.04

### BEKK model

The parameters A, B and C in the BEKK model are provided below:

$$ \begin{array}{l}\mathrm{A}=\left(\begin{array}{ccc}\hfill 0.40795405\hfill & \hfill 0.04365957\hfill & \hfill -0.0071803\hfill \\ {}\hfill 0.13445156\hfill & \hfill 0.93021649\hfill & \hfill 0.07466339\hfill \\ {}\hfill -0.9793208\hfill & \hfill -0.2015772\hfill & \hfill 0.01600388\hfill \end{array}\right),\;\mathrm{B}=\left(\begin{array}{ccc}\hfill 0.03700521\hfill & \hfill 0.02687299\hfill & \hfill 0.16010347\hfill \\ {}\hfill -0.0394133\hfill & \hfill -0.022465\hfill & \hfill 0.13606053\hfill \\ {}\hfill -0.003041\hfill & \hfill 0.00067659\hfill & \hfill -0.0419268\hfill \end{array}\right),\\ {}\;\mathrm{C}=\left(\begin{array}{ccc}\hfill 7.85009270\hfill & \hfill \hfill & \hfill \hfill \\ {}\hfill -0.0358002\hfill & \hfill 1.59068051\hfill & \hfill \hfill \\ {}\hfill 0.03869339\hfill & \hfill 0.20402223\hfill & \hfill 2.94049023\hfill \end{array}\right)\end{array} $$

**Figure 4:**
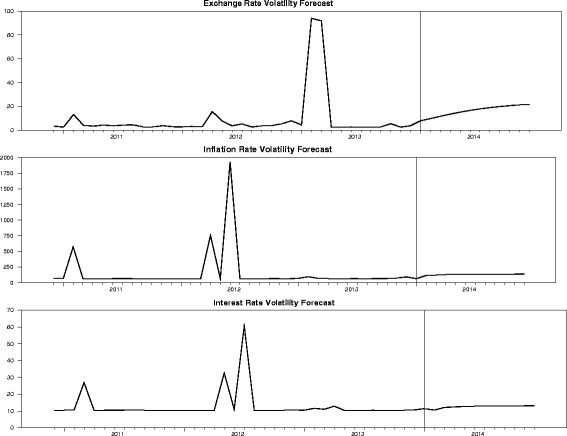
**Time series forecasts of volatility in inflation, exchange and interest rates.**

Figure 4 exhibits the time series forecast of volatility in inflation, exchange and interest rates for the next twelve months. The exchange rates forecast indicates that there is likely to be instability in the exchange rate in 2014. This implies that the cedi is likely to deviate abnormally in 2014, that is, the cedi is expected to depreciate very fast in 2014. The inflation rate forecast suggest that, in 2014, general prices of goods and services will increase but at a low rate, interest rates will also increase at the same pace. The forecasts suggest economic instability in Ghana in 2014. The shocks in the graph suggest that inflation and interest rates react to exchange rates volatility in the medium to long term. As at the time of completing this research work, the cedi has depreciated 31.8% on June 5, 2014, per information available on the Bank of Ghana website, a record high within the last decade, (Bank of Ghana, 2014). The current rate of 31.8% suggest that inflation rates could escalate further if the cedi is not stabilized by the last quarter of 2014.

Certainly, it is evident that the BEKK model is robust in modeling volatility in the depreciation of the cedi to other foreign currencies.

**Figure 5:**
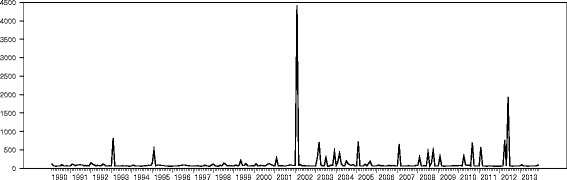
**Time series plot of inflation rate volatility from 1990 to 2013.**

Figure 5 displays time series plot of inflation rates volatility from 1990 to 2013. There is evidence of relatively mild volatility in 2004 and 2008. Volatility in inflation rate during the study period could be found in 1993, 1995, 2003, 2004, 2005, 2007, 2008, 2010, 2011 and 2012. It must be noted that, the highest shock was in 2002. The risk in inflation means that there is evidence of abrupt deviation from the mean of the general level of prices of goods and services. The volatility exhibited during these periods implies that the expected inflation deviated from the observed mean value. Inflation volatility measures the uncertainty in the expected inflation. Volatility of any kind is likely to deteriorate the prospects of a healthy economy, if volatility is high; investors become uncertain in their future investments since there is a high inflation risk, therefore demand a high return. High volatility in inflation leads to high cost of borrowing which directly affect investment negatively and to a large extent the health of the economy leading to ineffective planning. The trend in the plots indicates that inflation volatility trail exchange rate volatility; this suggests that, inflation reacts to exchange rate volatility in the long run.

**Figure 6:**
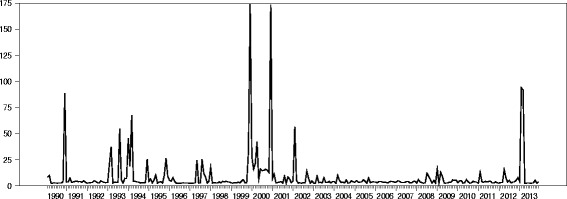
**Time series plot of exchange rate volatility from 1990 to 2013.**

Figure 6 is a time series plot of exchange rate volatility from 1990 to 2013. The period between 2002 and 2012 exhibited relatively mild deviation in mean exchange rate suggesting stability. Much of the turbulence could be observed between 2001 and 1990 as well as in 2013. The plot seems to suggest that exchange rate exhibits some sort of shocks a year after the general presidential and parliamentary elections are held in Ghana. It also suggests that the cedi depreciates fast during the first quarter of every year. The shocks in exchange rate impacts negatively on the economy of Ghana since it weakens the Ghanaian cedi against the US dollar. Volatility in the exchange rate will result in high prices of imported goods and services and reduces investor confidence in the economy. This implies that there will be uncertainty in the expectation of how the cedi will perform on the forex, as such many are likely to speculate, the public react by demanding more dollars, all things being equal, the cedi will depreciate further. The gross domestic product, employment and the overall health of the economy of Ghana will be affected negatively as a result.

### Vector error correction model and granger causality

The Vector Error Correction Model and Granger Causality test is used to examine the cause and effect of the inflation rate, exchange rate and interest rate. Johansen test of cointegration among the variables using STATA 12 rejected the null hypothesis that there is no cointegration; a precondition to running the Vector Error Correction model as shown in Table 3.

**Table 3:** **Johansen test of cointegration among the variables using STATA 12**

**Maximum rank**	**Eigen values**	**Trace statistics**	**5% critical value**
0		29.73	29.68
1	0.06181	11.6746	15.41
2	0.03089	2.7963	3.76
3	0.00983		

The Vector Correction Model evidence long run and short run causality among the variables after the null hypothesis of both “no long run causality and no short run causality” were rejected. After a pair-wise Granger-causality tests at 5% significant level, the result show that, exchange rate Granger-cause inflation rate but the converse does not. Similarly, inflation rate Granger cause interest rate but the reverse does not.

## Conclusions

Multivariate GARCH, DCC and BEKK models were fitted to the variances of the data. Both models passed the diagnostic test. The mean equation of the DCC model was used to predict the expected inflation rate and proved to be robust in the short to medium term, similarly, the BEKK model was used to predict the expected exchange rate volatility.

These predictions suggest that, inflation rates are expected to increase at a very slow rate in 2014. Also, the forecast of exchange rate volatility suggested that, there is a very high risk of abrupt depreciation of the cedi to the US dollar. This implies that the rates of inflation as well as interest rates are likely to react in the long run to the expected volatility in exchange rate for the year 2014.

There was generally positive conditional and unconditional correlation between inflation rates and exchange rates, inflation rates and interest rates as well as exchange rates and interest rates. This implies that there is some evidence that when the general prices of goods and services are stable, interest rates are expected to be stable and possibly low. That of inflation and exchange rates implies that the stability of inflation means that the cedi depreciated to the dollar at low rate. There was evidence that the cedi has depreciated cumulatively to the US dollar of 7010.02% from 1990 to 2013 with a weighted annual average depreciation of 20.4%.

The volatility experienced in inflation, exchange and interest rates in the study, to a large extent were not in elections year. It is therefore factually inaccurate to assert that during election years, the cedi depreciates faster to the US dollar. The evidence rather suggests, there seem to be volatility in these economic variables, periods after elections were held rather than during elections year and also during the first quarter of every year.

It was also evident that, the fact that inflation rates were stable, does not mean that exchange rates and interest rates are expected to be stable. Rather, when the cedi performs well on the forex, inflation and interest rates react positively in the long run. All things being equal, this reaction tickles down to all aspects of the economy thus, occasioning improved standards of living.

The economy of Ghana reacts positively in most instances when the cedi performs strongly on the forex market. Such performance was evidenced in 2003 when the cedi depreciated to the US dollar at an average of 3.81%, during that same year the Ghana Stock Exchange recorded returns on investments of about 155%, the highest since its inception. The success of the cedi during this year could be traced to foreign inflows of HIPC benefits into the country. This implies that the health of the economy of Ghana is highly dependent on the strength of the cedi against foreign currencies such as the US dollar, Euro and the British pound sterling.

### Recommendations

Recommendations are made for both policy formulation and areas of further research based on the findings of the study.

To begin with, it is recommended that policy makers use multivariate GARCH models to study the dynamics of economic and financial data. The DCC model proved to be robust in modeling the correlation among inflation, exchange and interest rates, and the mean equation of the model was robust for modelling inflation rates in the short to medium term. Similarly, the BEKK model was found to be robust in modeling volatility as well as forecasting.

Secondly, the research work has revealed that, the health of Ghana’s economy is highly dependent on the strength of the Ghanaian currency: cedi against the foreign currencies since the country is import dependent, as such there must be a national agenda to increase foreign inflows and introduce a policy aimed at Exchange Rate Targeting (ERT). The forecast is also an indication that policy makers and industry players can effectively plan to curb uncertainties in the Ghanaian economy given these models are used.

Thirdly, there must be a national consensus to reduce imports into the country by improving production and in the long run increase non-traditional exports. The government could adopt a policy through consensus with private sectors (services) to list on the Ghana Stock Exchange to attract Ghanaians to own shares, tax incentives could be used as a stimulus package. This is to ensure that 100% of the profit is not repatriated. Government could also dialogue with the private sector and propose a policy that mandates foreign owned companies to delay about 50% repatriation of their profit in the economy of Ghana for about two years. Government must also adopt a policy to reduce the number of State delegations to international events abroad to about 20%, this could also reduce the pressure on the Ghanaian cedi.

Lastly, a study into the dynamics of interest rates, stock returns and exchange rates is recommended. Other economic indicators such as money supply, balance of payment and budget deficit could be added to inflation rate, exchange rate and interest for modelling using multivariate GARCH models. Modelling the volatility in the five most traded currencies in Ghana is also recommended. Impulse analysis of inflation rates, exchange rates and interest rates is suggested as well.
